# Short-Chain Fatty Acids Inhibit Growth Hormone and Prolactin Gene Transcription via cAMP/PKA/CREB Signaling Pathway in Dairy Cow Anterior Pituitary Cells

**DOI:** 10.3390/ijms141121474

**Published:** 2013-10-30

**Authors:** Jian-Fa Wang, Shou-Peng Fu, Su-Nan Li, Zhong-Ming Hu, Wen-Jing Xue, Zhi-Qiang Li, Bing-Xu Huang, Qing-Kang Lv, Ju-Xiong Liu, Wei Wang

**Affiliations:** 1 College of Veterinary Medicine, Jilin University, Changchun 130062, China; E-Mails: wjflw@sina.com (J.-F.W.); shoupengfu@163.com (S.-P.F.); lsnlhh2006@163.com (S.-N.L.); zhulingxue_1991@163.com (W.-J.X.); w120658200@163.com (Z.-Q.L.); huangbingxu123@gmail.com (B.-X.H.); lvqingkang@hotmail.com (Q.-K.L.); 2 College of Animal Science and Veterinary Medicine, Heilongjiang Bayi Agricultural University, Daqing 163319, China; 3 Laboratory of the Animal Center, Academy of Military Medical Sciences, Beijing 100850, China; E-Mail: huzhongming2005@sina.com

**Keywords:** short-chain fatty acids, dairy cow, growth hormone, prolactin, G protein

## Abstract

Short-chain fatty acids (SCFAs) play a key role in altering carbohydrate and lipid metabolism, influence endocrine pancreas activity, and as a precursor of ruminant milk fat. However, the effect and detailed mechanisms by which SCFAs mediate bovine growth hormone (*GH*) and prolactin (*PRL*) gene transcription remain unclear. In this study, we detected the effects of SCFAs (acetate, propionate, and butyrate) on the activity of the cAMP/PKA/CREB signaling pathway, *GH*, *PRL*, and *Pit-1* gene transcription in dairy cow anterior pituitary cells (DCAPCs). The results showed that SCFAs decreased intracellular cAMP levels and a subsequent reduction in PKA activity. Inhibition of PKA activity decreased CREB phosphorylation, thereby inhibiting *GH* and *PRL* gene transcription. Furthermore, PTX blocked SCFAs- inhibited cAMP/PKA/CREB signaling pathway. These data showed that the inhibition of *GH* and *PRL* gene transcription induced by SCFAs is mediated by Gi activation and that propionate is more potent than acetate and butyrate in inhibiting *GH* and *PRL* gene transcription. In conclusion, this study identifies a biochemical mechanism for the regulation of SCFAs on bovine *GH* and *PRL* gene transcription in DCAPCs, which may serve as one of the factors that regulate pituitary function in accordance with dietary intake.

## 1. Introduction

The pituitary plays a critical role in animals and regulates a broad range of physiological processes involved in growth, metabolism, reproduction, lactation, and stress [1]. Growth hormone (GH) is a polypeptide hormone synthesized and secreted by the anterior pituitary gland. It plays a key role in regulating ruminant mammary gland development and lactation [2]. In lactating cows, bovine GH induces proliferation of mammary parenchyma and growth of epithelial cells, and increase the cell renewal in the mammary gland [3]. GH increases milk protein gene expression in bovine mammary explants and mammary epithelial cells [4,5]. Prolactin (PRL) is a polypeptide hormone that is synthesized in and secreted from lactotrophs of the anterior pituitary gland [6]. PRL also play a key role in regulating mammary gland development and lactation [7]. Because of the irreplaceable regulatory role in growth, metabolism, and lactation, researches on endocrinology always focus on the factors and mechanisms affecting GH and PRL synthesis and release.

Short-chain fatty acids (SCFAs), including acetate, propionate, and butyrate, are formed during bacterial fermentation of carbohydrates in the gut or rumen of various animal species [8]. Dietary nutrients play an important role in regulating rumen fermentation and SCFAs synthesis. The rumen ratio of acetate to propionate to butyrate is approximately 70:20:10 in lactating dairy cows, and the mean concentrations of plasma acetate, propionate, and butyrate are 0.4, 0.1, and 0.5 mmol/L in dairy cows [9]. SCFAs have multiple *in vivo* and *in vitro* effects including mediate inflammation, alter carbohydrate and lipid metabolism, regulate cell proliferation and differentiation, influence endocrine pancreas activity, provide an additional source of energy for the body, and as a precursor of ruminant milk fat [10–13]. In addition to these, SCFAs are known to have *in vivo* and *in vitro* actions on pituitary hormone secretion function. Addition of sodium-butyrate to milk formula increased the secretion of GH and insulin level in pre-weaning calves [14]. The sodium salts of butyric, valerate, hexanoic, caprylic, nonanoic, and dodecanoic acids increased GH and prolactin (PRL) secretion in GH3 cell [15]. By contrast, the reported effects of SCFAs on GH secretion are still controversial. Ishiwata *et al.* found that addition of propionate or butyrate to the anterior pituitary cells of the goat cultured *in vitro* inhibited GHRH-induced GH release and GH production [16]. Therefore, the effect and detailed mechanisms by which SCFAs mediate bovine pituitary function need to be elucidated.

In 2003, two orphan G protein coupled receptors (GPCRs), GPR41 and GPR43 have been identified as cell-surface receptors for SCFAs [17]. Both GPR41 and GPR43 are coupled with G_q_ and G_i/o_, and their activation can induce an increase in intracellular calcium concentration and suppress cellular cyclic adenosine 3′,5′-monophosphate (cAMP) accumulation [18]. Wang *et al.* has proved that *GPR41* and *GPR43* mRNA are expressed in bovine pituitary gland [19]. Pituitary-specific positive transcription factor 1 (Pit-1) was first discovered as the transcription factor that is necessary for the expression of *GH* and *PRL* [20]. The proximal promoters of the rat *GH* gene include binding sites for Pit-1, specificity protein 1 (Sp1), cAMP-response element binding protein (CREB), and thyroid hormone response element (TRE) [21,22]. The promoters of the rat *PRL* gene include binding sites for Pit-1, estrogen response element (ERE), and Ets binding sites (EBS) [6]. The *Pit-1* promoter contains a binding site for Pit-1 and two CREB binding sites [23]. Thus, the change of phosphorylation levels of CREB could change *GH* and *PRL* gene transcription level directly or indirectly. We hypothesize that SCFAs may mediate bovine *GH* and *PRL* gene transcription via the G protein signaling pathway. Therefore, the objective of this study was to determine the effects of SCFAs on the activity of G protein signaling pathway, *GH*, *PRL*, and *Pit-1* gene transcription in dairy cow anterior pituitary cells (DCAPCs). The results of this study could provide important information for understanding the role of the G protein signaling pathway in SCFAs mediate bovine pituitary function.

## 2. Results

### 2.1. Effect of SCFAs on mRNA Levels of GH, PRL and Pit-1 in DCAPCs

**Figure 1:**
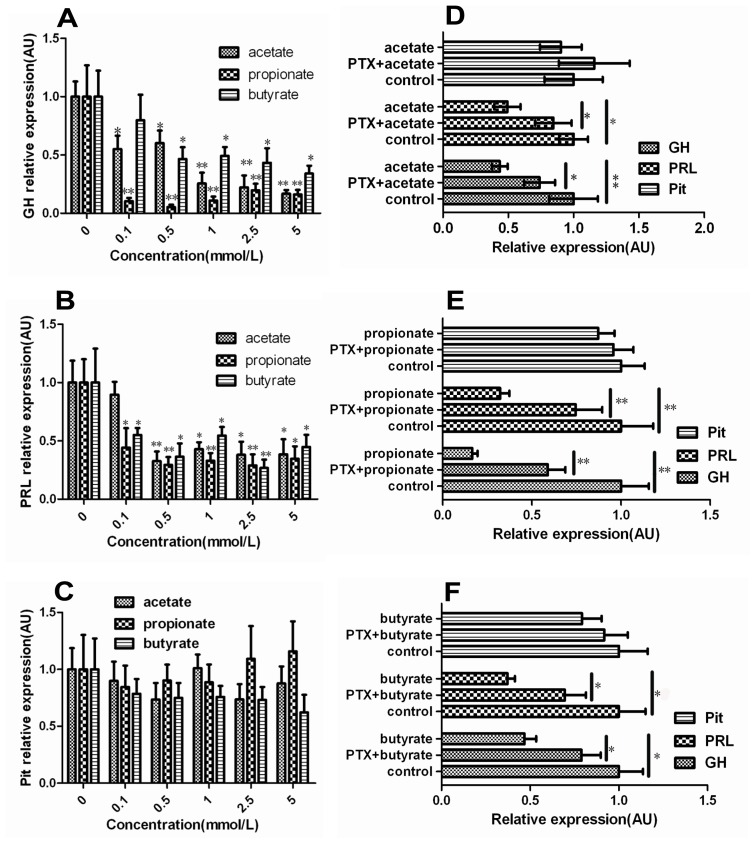
The effect of short-chain fatty acids (SCFAs) on the mRNA levels of *GH*, *PRL* and *Pit-1* in dairy cow anterior pituitary cells (DCAPCs). DCAPCs were treated with 0, 0.1, 0.5, 1.0, 2.5 and 5.0 mmol/L acetate (**A**); propionate (**B**); and butyrate (**C**) for 24 h, respectively. The cells were also treated with or without prior PTX incubation (100 ng/L) for 2 h, and then stimulated with 1.0 mmol/L acetate (**D**); propionate (**E**); or butyrate (**F**) for 24 h, respectively. Each treatment replicated 12 times. * *p* < 0.05, ** *p* < 0.01 *vs.* the control group.

The mRNA levels of *GH* and *PRL* showed a decreasing trend in the SCFAs-treated groups. The mRNA levels of *GH* were significantly lower in the 0.1 and 0.5 mmol/L acetate and 0.5, 1.0, 2.5 and 5.0 mmol/L butyrate groups than in the control groups (Figure 1A; *p* < 0.05), and the mRNA levels of *GH* were markedly lower in the 1.0, 2.5 and 5.0 mmol/L acetate and 0.1, 0.5, 1.0, 2.5 and 5.0 mmol/L propionate groups than in the control groups (Figure 1A; *p* < 0.01), respectively. The mRNA levels of *PRL* were significantly lower in the 1.0, 2.5 and 5.0 mmol/L acetate, 0.1 and 5.0 mmol/L propionate and 0.1, 0.5, 1.0 and 5.0 mmol/L butyrate groups than in the control groups (Figure 1B; *p* < 0.05), and the mRNA levels of *PRL* were markedly lower in the 0.5 mmol/L acetate, 0.5, 1.0, 2.5 mmol/L propionate and 1.0 and 2.5 mmol/L butyrate groups than in the control groups (Figure 1B; *p* < 0.01), respectively. The transcription of the *Pit-1* gene had no obvious change treated by either acetate, propionate or butyrate at 0.1, 0.5, 1.0, 2.5 and 5.0 mmol/L (Figure 1C; *p* > 0.05), respectively. The transcription of the *GH* and *PRL* gene were significantly higher in the PTX + acetate group than in the 1.0 mmol/L acetate treatment group (Figure 1D; *p* < 0.05). The mRNA levels of *GH* and *PRL* were markedly higher in the PTX + propionate group than in the propionate treatment group (Figure 1E; *p* < 0.01). The mRNA levels of *GH* and *PRL* were significantly higher in the PTX + butyrate group than in the butyrate treatment group (Figure 1E; *p* < 0.01). Taken together, these results strongly suggest that SCFAs significantly down-regulate the expression of *GH* and *PRL* gene in DCAPCs, and the mRNA levels of *GH* and *PRL* were significantly higher by prior PTX incubation.

### 2.2. Effect of SCFAs on Intracellular cAMP Concentration

**Figure 2:**
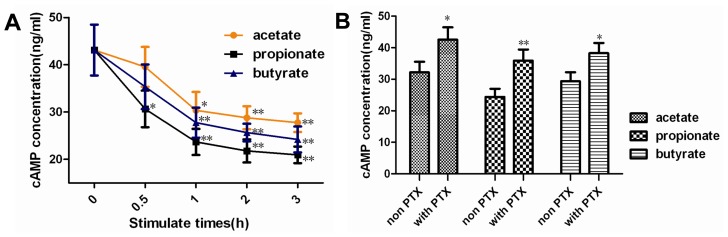
The effect of SCFAs on the intracellular cAMP levels in DCAPCs. DCAPCs were treated with 1.0 mmol/L acetate, propionate, and butyrate for 0, 0.5, 1.0, 2.0, and 3.0 h, respectively (**A**); The cells were also treated with or without prior PTX incubation (100 ng/L) for 2 h, and then stimulated with 1.0 mmol/L acetate, propionate, or butyrate for 3 h, respectively (**B**). Each treatment replicated 12 times. * *p* < 0.05, ** *p* < 0.01 *vs.* the control group.

The cAMP levels were lower in the SCFAs-treated groups than in the control group, and both were the lowest at the 3 h of the SCFAs-treated groups (Figure 2A). The cAMP levels were significantly higher in the PTX + acetate or butyrate group than in the non PTX treatment group (Figure 2B; *p* < 0.05), respectively. The cAMP level was significantly higher in the PTX + propionate group than in the non PTX treatment group (Figure 2B; *p* < 0.01). Overall, these results indicate that SCFAs significantly decrease the intracellular cAMP concentration, and the intracellular cAMP levels were significantly decreased by prior PTX incubation.

### 2.3. Effect of SCFAs on PKA Activity

**Figure 3:**
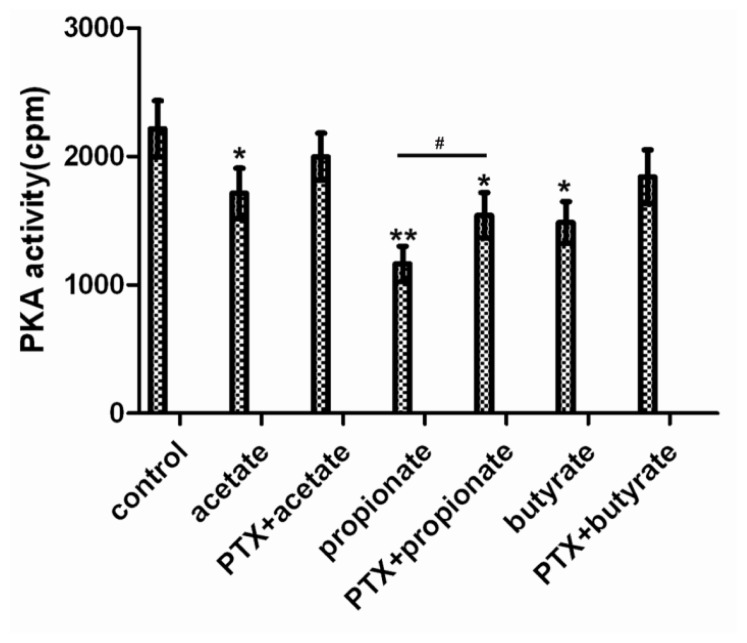
The effect of SCFAs on the PKA activity in DCAPCs. The cells were treated with or without prior PTX incubation (100 ng/L) for 2 h, and then stimulated with 1.0 mmol/L acetate, propionate, or butyrate for 3 h, respectively. Each treatment replicated 12 times. *p < 0.05, **p < 0.01 *vs.* the control group; # *p* < 0.05 *vs.* the non PTX group.

The PKA activity was lower in the SCFAs-treated groups than in the control group, and the inhibiting effect of SCFAs was blocked by PTX (Figure 3). The PKA activity was significantly lower in the acetate and butyrate group than in the control group (*p* < 0.05), respectively. The PKA activity was markedly lower in the propionate group than in the control group (*p* < 0.01). The PKA activity was significantly increased in the PTX + propionate group than non PTX group (*p* < 0.05). The PKA activity had no obvious change in the PTX + acetate or butyrate group than non PTX treatment group (*p* > 0.05), respectively.

### 2.4. Effect of SCFAs on CREB Phosphorylation

**Figure 4:**
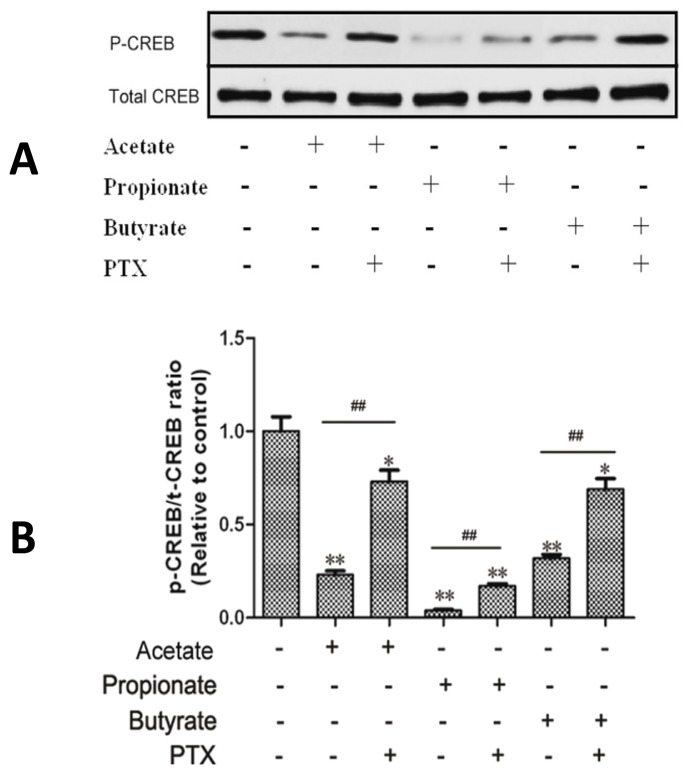
The effect of SCFAs on the CREB phosphorylation in DCAPCs. The cells were treated with or without prior PTX incubation (100 ng/L) for 2 h, and then stimulated with 1.0 mmol/L acetate, propionate, or butyrate for 6 h, respectively. Each treatment replicated 12 times. * *p* < 0.05, ** *p* < 0.01 *vs.* the control group; ## *p* < 0.01 *vs.* the non PTX group.

The phosphorylation levels of CREB (Figure 4) were markedly lower in the acetate-, propionate- and butyrate-treated groups than in the control group respectively (*p* < 0.01), and the phosphorylation levels of CREB were markedly higher by prior PTX treatment (*p* < 0.01).

## 3. Discussion

SCFAs, primarily acetate, propionate and butyrate, are organic acids produced within the intestinal lumen or rumen of ruminant by bacterial fermentation of mainly undigested dietary carbohydrates [24]. There has been increasing evidence that the majority of SCFAs play an essential role in maintaining the health of colonic mucosa [25–28]. Due to the important role of SCFAs, researchers are interested in finding the methods to increase intestinal SCFAs concentrations [29,30]. In contrast to the wide range of positive effects of SCFAs on the health, few studies have reported the adverse effects of SCFAs. SCFAs are known to have *in vivo* and *in vitro* actions on pituitary secretion function [14–16]. However, the effect and detailed mechanisms by which SCFAs mediate bovine *GH* and *PRL* gene transcription remain unclear. More emphasis should be put on *GH* and *PRL* gene transcription regulation to elucidate the role of the G protein signaling pathway in SCFAs mediate bovine pituitary function.

GPCRs are cell surface receptors which serve in the transduction of extracellular stimuli into intracellular signals. They mediate extracellular stimuli into intracellular signals and are indispensable among membrane proteins because they constitute the largest and most diverse groups of receptor proteins. Thus, GPCRs represent one of the most important families of drug targets. The regulatory subunit of G proteins has four classes: G_αs_, G_αi_, G_αq_, and G_α12_ with respect to sequence homology and functional similarities of their α subunits [31]. The activation of G_αi_ results in an inhibition of adenylyl cyclase, hence, decrease in cAMP production [32]. In this study, the cAMP levels were significantly lower in the SCFAs-treated groups than control group. ADP-ribosylation of the G_αi_-proteins prevents the coupling to their cognate GPCRs and consequently disrupts the signal transduction cascade. Here, we demonstrated that PTX could inhibit SCFAs-induced decrease in cAMP level. This indicates that SCFAs exert their actions through coupled to G_αi_ subunit. The biological effects of PTX that result from ADP-ribosylation of the G_αi_-proteins are diverse, some of which can be attributed to the pretreatment timing and load dosing. Almost all cell types G_αi_-protein were nearly completely ADP-ribosylated after a 2 h incubation with PTX (200 ng/mL) [33]. In most cells, PTX itself only induces an incremental increase in cAMP when the load dosing reach more than 1 μg/mL [33]. Therefore, PTX (100 ng/L) could not restore the effect of SCFA to the control level in DCAPCs, some of which may be attributed to the load dosing of PTX.

Decrease in cAMP levels results in a subsequent reduction in PKA activity. Then, PKA catalyzes the phosphorylation of serine residues on CREB to activate pit-1 and thus initiate a response within the cell [34]. Our data showed that the PKA activity was lower in the SCFAs-treated groups than in the control group, and the inhibiting effect of SCFAs was blocked by PTX. Moreover, the phosphorylation levels of CREB were lower in the SCFAs-treated groups than in the control group, and this effect was also decreased by prior PTX incubation. Overall, these results indicate that SCFAs could inhibit the activity of cAMP/PKA/CREB signaling pathway in DCAPCs.

In the nucleus, phosphorylated CREB could change *GH* gene transcription level directly or activate Pit-1 to trigger the transcription of the *GH* and *PRL* gene indirectly [34]. In this study, SCFAs significantly down-regulate the expression of *GH* and *PRL* gene in DCAPCs, and the mRNA levels of *GH* and *PRL* were significantly higher by prior PTX incubation. Based on these observations, we conclude that SCFAs inhibit *GH* and *PRL* gene transcription via cAMP/PKA/CREB signaling pathway.

However, the expression of *Pit-1* gene was not significantly changed, suggesting that the change of Pit-1 activity may play an important role in the regulation of *GH* and *PRL* transcription by SCFA. Three serine-threonine phosphorylation sites have been identified in Pit-1:serine 115 (S115), threonine 219 (T219), and threonine 220 (T220). The S115 and T220 sites are phosphorylated both in cells treated with cAMP or phorbol esters or by directly phosphorylating purified Pit-1 proteins with PKA and PKC [35–37]. Phosphorylation of Pit-1 at both T220 and S115 is elevated in mitotic cells and binding of Pit-1 to the PRL-_1p_, GHF-_1p_, and GH-_1p_ sites was reduced [37]. Thus, we hypothesize that phosphorylated CREB may not mediate bovine *PRL* gene transcription via increase the phosphorylation levels of Pit-1. The CREB binding protein (CBP) acts as a cofactor for Pit-1-dependent activation of the hGH promoter by the GHRH signaling pathway and PKA [38]. CBP acts by binding to phosphorylated CREB (PKA-dependent) and activating gene expression [39]. For these reasons, phosphorylated CREB may increase the phosphorylated CBP or CBP complex interacts with Pit-1 resultant activation of transcription of the bovine *PRL* gene. Thus, further studies are needed to understand the exact mechanisms mediating bovine *PRL* gene by CREB.

SCFAs are the major energy source for dairy cows, which promote rumen development in calves and rumen epithelial papilla growth in lambs [40]. Zhao and Sun found that the concentration of GH in plasma was increased significantly with increasing level of ruminal infusion of mixed SCFAs [41]. Kato *et al.* found that addition of sodium-butyrate to milk formula increased the secretion of GH and insulin level in pre-weaning calves [14]. However, Matsunaga *et al.* found that ruminal or mesenteric venous infusion of SCFAs significantly suppressed GH secretion in a dose-dependent manner [42–44]. Factors affecting GH or PRL synthesis and secretion in the dairy cow include hypothalamic factors, peripheral hormones, cytokines, and dietary nutrients [6,45]. Thus, there was a very large difference results among *in vivo* experiments according to the difference physiological state of the animal. Furthermore, the network integrating hormone, neurotransmitters, cytokines, and nutrient signaling in control of GH and PRL synthesis and secretion is complex and make interpretation difficult. It seems, therefore, that existing *in vivo* approaches are not effective for the investigation the effect and exact mechanisms of SCFAs on the GH and PRL synthesis and secretion in the dairy cows. Cell culture is one of the most convenient tools for understanding the mechanisms of pituitary hormone synthesis and secretion, which provides a powerful tool to uncover new information. Based on these reasons, we established a dairy cow GH and PRL secreting anterior pituitary cell model, which can be used successfully for the study of the mechanisms of hypothalamic factors, peripheral hormones, cytokines, or dietary nutrients regulating GH or PRL synthesis and release [46]. Mean acetate, propionate, and butyrate level in cerebrospinal fluid (CSF) of cattle are lower than in plasma [47,48]. In this study, a wide range concentration of SCFAs, which include the physiology concentration of SCFAs in CSF and plasma, was chosen. This study identifies a biochemical mechanism for the regulation of SCFAs on bovine *GH* and *PRL* gene transcription in DCAPCs, which may serve as one of the factors that regulate pituitary function in accordance with dietary intake.

## 4. Experimental Section

### 4.1. Materials

Fetal bovine serum (FBS), bovine serum albumin (BSA), trypsogen, and DMEM medium were purchased from Gibco (Grand Island, NY, USA). Sodium-acetate, sodium-propionate, sodium-butyrate, pertussis toxin (PTX), hyaluronidase, Dnase, and I type collagenase were provided by the Sigma Aldrich (St. Louis, MO, USA). Cell culture plates were purchased from Corning Incorporated (Corning, New York, USA). Bovine cAMP assay kit was purchased from R & D Systems (Minneapolis, MN, USA). Anti-phospho CREB (Ser^133^), anti-CREB antibodies, and PKA assay kit were purchased from Millipore (Billerica, MA, USA).

### 4.2. Isolation and Culture of DCAPCs

Pituitary glands were extracted from the heads of Holstein cows by electric saw at a local slaughterhouse and transported to the laboratory in HBSS solution without calcium and magnesium (CMF-HBSS) to which was added 50 μg streptomycin/mL, and 100 IU penicillin/mL at 5 °C within 2 h. The posterior pituitary was removed with precision medical scissors and tweezers to obtain integrated anterior pituitary glands. Anterior pituitary glands were located were diced into small pieces less than 1 mm^3^ and incubated in CMF-HBSS containing 0.3% I type collagenase, 0.1% hyaluronidase and 0.1‰ DNase at 37 °C for 2 h. The dispersed cells were washed three times with HBSS, resuspended in DMEM medium supplemented with 10% fetal bovine serum at seeding density of 1 × 10^3^ cells/mL. Then, the cells were seeded into a 75 cm^2^ culture flask and incubated at 37 °C in a humidified atmosphere containing 5% CO_2_.

### 4.3. Real-Time RT-PCR

For real-time PCR analysis, DCAPCs were grown in 6-well plates (5 × 10^5^ cells), serum starved for 24 h, and then stimulated with 0, 0.1, 0.5, 1.0, 2.5, and 5.0 mmol/L acetate, propionate, or butyrate for 24 h. PTX is the ADP-ribosylating toxin produced by the whooping cough causing bacterium *Bordetella pertussis*. ADP-ribosylation of the α subunit of heterotrimeric G_αi_ proteins locks the α subunits into an inactive state, thus it is unable to inhibit adenylyl cyclase [49]. It is widely applied as a tool in biochemical and pharmacological studies for the investigation of signaling pathways involving heterotrimeric G proteins [33]. DCAPCs were also treated with or without prior PTX incubation (100 ng/L) for 2 h, and then stimulated with 1.0 mmol/L acetate, propionate, or butyrate for 24 h. Total RNA was isolated from cells with trizol reagent (Invitrogen, Carlsbad, CA, USA) according to the manufacturer’s instructions. Total RNA was treated with DNase I (Takara, Kyoto, Japan) to remove genomic DNA contamination. Total RNA concentration and purity was determined using a spectrophotometer (Bio-Rad, Hercules, CA, USA). Only samples with an optical density ratio at 260/280 nm 1.8–2.2 were used in further analysis. Total RNA integrity was checked by electrophoresis on an agarose gel. Total RNA samples were reverse transcribed using a reverse transcription kit (Takara, Kyoto, Japan) according to the manufacturer’s instructions. The primer sequences of *GH*, *PRL*, *Pit-1*, and *GAPDH* genes (Table 1) were designed as Laporta *et al.* described [50]. *GH*, *PRL*, and *Pit-1* expression were evaluated by quantitation RT-PCR analysis using the Premix Ex Taq™ (Takara, Kyoto, Japan). The relative expression level for *GH*, *PRL*, and *Pit-1* was calculated relative to *GAPDH* (the normalizer) using the comparative cycle threshold method [51,52].

### 4.4. Measurement of Intracellular cAMP Concentration

DCAPCs were grown in 6-well plates (5 × 10^5^ cells), serum starved for 24 h, and then stimulated with 1.0 mmol/L acetate, propionate, or butyrate for the assigned period (0.5, 1, 2, 3 h). The cAMP-dependent pathway is a GPCR-triggered signaling cascade. Thus, the intracellular cAMP was extracted from the cells and the concentration was assayed by microplate reader (Bio-Rad, Hercules, CA, USA) using rat cAMP assay kit according to the manufacturer’s instructions. DCAPCs were also treated with or without prior PTX incubation (100 ng/L) for 2 h, and then stimulated with 1.0 mmol/L acetate, propionate, or butyrate for 3 h. The assay is based on the competition between unlabeled cAMP and a fixed quantity of horseradish peroxidase (HRP)-labeled cAMP for a limited number of binding sites of a cAMP specific antibody.

### 4.5. Measurement of PKA Activity

cAMP works by activating protein kinase A (PKA), then, leads to the phosphorylation of cAMP response element binding protein (CREB). Thus, the PKA activity and phosphorylation levels of CREB were determined in this study. DCAPCs were cultured in the presence or absence of acetate, propionate, or butyrate (1.0 mmol/L) in FBS-F12 for 3 h. The cells were also treated with prior PTX incubation (100 ng/L) for 2 h compared with groups without prior PTX incubation. Activity of PKA was determined using a radioactive method using PKA assay kit according to the manufacturer’s instructions. Briefly, the lysates were incubated for 30 min at 30 °C with 100 μm PKA-specific substrate, and 10 μCi of [γ-^32^P] ATP in kinase buffer, supplemented or not with PKA inhibitor peptide. The phosphorylated substrate was quantitated by using a liquid scintillation counter (Beckman coulter, Brea, CA, USA).

### 4.6. Western Blotting Analysis of Phosphorylated CREB

DCAPCs were grown in 6-well plates (5 × 10^5^ cells), serum starved for 24 h, and then stimulated with 1.0 mmol/L acetate, propionate, or butyrate for 6 h. Cells were also treated with prior PTX incubation (100 ng/L) for 2 h compared with groups without prior PTX incubation. Nuclear proteins were extracted by nuclear protein extraction kit according to the manufacturer’s instructions (Beyotime Co., Nantong, China). Protein concentrations were determined by bicinchoninic acid (BCA) protein assay kit (Beyotime Co., Nantong, China). Aliquotes of cell lysates containing 20 μg protein were separated in 10% polyacrylamide gels, electrophoretically transferred to PVDF membranes (Millipore, Billerica, MA, USA) using Bio-Rad criterion blotter (Bio-Rad, Hercules, CA, USA). Membranes were blocked in 5% non-fat, dried milk in TBST (50 mmol/L Tris, pH 7.5, 0.15 mol/L NaCl, 0.05% Tween-20) at room temperature for 1 h and then incubated with either anti-phospho CREB (1:3000 dilution) or anti-CREB (1:2000 dilution) at 4 °C overnight. Blots were then washed and incubated with horseradish peroxidase-labeled secondary antibodies (Sigma Aldrich, St. Louis., MO, USA) at 37 °C for 1 h. Immunoreactive bands were detected with enhanced chemiluminescence (ECL) western blotting detection reagents (Beyotime Co., Nantong, China). Blots were exposed to X-ray film for radiography of the bands. Blots were digitally detected and measured using a LAS3000 Bioimage Analyzer (Fuji Photo Film, Tokyo, Japan).

### 4.7. Statistical Analyses

Results were expressed as means ± SD. Data were analyzed by using statistical software package SPSS 12.0 (SPSS Inc, Chicago, IL, USA). Groups were compared by one-way analysis of variance (ANOVA) followed by the least significant difference test. A *p* value of less than 0.05 was considered statistically significant, and values less than 0.01 were considered markedly significant.

## 5. Conclusions

**Figure 5:**
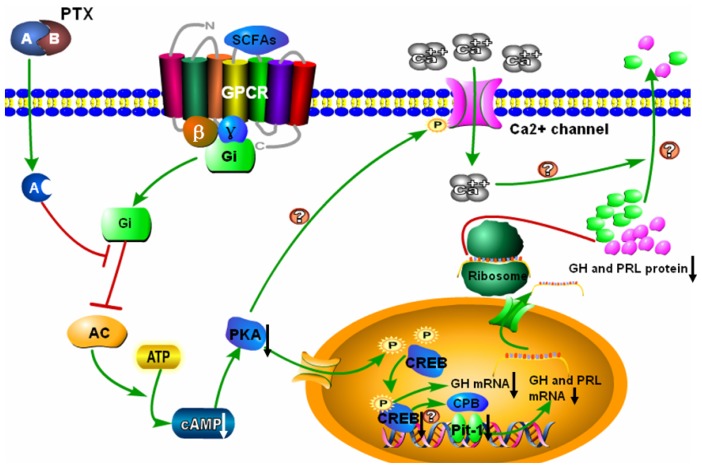
SCFAs inhibit the cAMP/PKA/CREB signaling pathway to mediate *GH* and *PRL* gene transcription in DCAPCs. SCFAs bind to GPCR and lead to dissociation of heterotrimeric G protein complex into G_αi_ and G_βγ_ subunit. The exchange of GTP from GDP results in activation of the G_αi_, thereby inhibiting adenylyl cyclase (AC) activity. This process results in a decrease of intracellular cAMP levels and a subsequent reduction in PKA activity. Inhibition of PKA activity inhibits CREB phosphorylation, thereby decreasing *GH* and *PRL* gene transcription directly or indirectly. The A-protomer of PTX penetrates into the host cells result in inactivation of G_αi_, hence, inhibits SCFAs mediated signaling pathway. In addition, inhibition of PKA activity may result in a decreased of Ca^2+^ influx through decreased l-type Ca^2+^ channel mean open time. Therefore, SCFAs may also inhibit bovine GH and PRL secretion via G_αi_-mediated inhibition of Ca^2+^ channel.

In summary, the results of this study indicate that SCFAs, as signaling molecules, significantly decrease *GH* and *PRL* gene transcription in DCAPCs, putatively according to the mechanism shown graphically in Figure 5. SCFAs bind to GPCR and lead to dissociation of heterotrimeric G protein complex into G_αi_ and βγ subunit. The exchange of GTP from GDP results in activation of the G_αi_, thereby inhibiting adenylyl cyclase activity. Inactivated adenylyl cyclase results in a decrease of intracellular cAMP levels and a subsequent reduction in PKA activity. Inhibition of PKA activity inhibits CREB phosphorylation, thereby leading to a decrease of bovine *GH* gene transcription. The change of the phosphorylation levels of CREB may decrease the phosphorylated CBP or CBP complex interacts with Pit-1 resultant inhibition of transcription of the bovine *PRL* gene. Consequently, SCFAs inhibit bovine *GH* and *PRL* gene transcription in DCAPCs. A-protomer of PTX penetrates into the host cells result in inactivation of G_αi_, hence, inhibits SCFAs mediated signaling pathway. In addition, inhibition of PKA activity may result in the decreased of Ca^2+^ influx through decreased l-type Ca^2+^ channel mean open time [53]. Therefore, SCFAs may also inhibit bovine GH and PRL secretion via G_αi_-mediated inhibition of Ca^2+^ channel. This study identifies a biochemical mechanism for the regulation of SCFAs on *GH* and *PRL* gene transcription in DCAPCs, which may serve as one of the factors that regulate bovine pituitary function in accordance with dietary intake. Future studies will further research the role and mechanism of SCFAs in regulating bovine pituitary hormone secretion.

## Figures and Tables

**Table 1:** The primer sequences of bovine *GAPDH*, *GH*, *PRL*, and *Pit-1*.

Gene	Sequences	Length (bp)
*GAPDH*	(F)5′-TGCCCAGAATATCATCCC-3′ (R)5′-AGGTCAGATCCACAACAG-3′	134
*GH*	(F)5′-AGATCCTCAAGCAGACCTA-3′ (R)5′-AGGTACGTCTCCGTCTTA-3′	121
*PRL*	(F)5′-TATGAAAGGAGCCCCAGATG-3′ (R)5′-CACACAGGGTAGGGCTCAGT-3′	137
*Pit-1*	(F)5′-TTCTGCAACTCTGCCTCTGA-3′ (R)5′-CCATAGGTCGATGACTGGT-3′	148
